# Rheological Investigation of Polydimethylsiloxane with Glass Beads: A Model for Compression-Stiffening Effects in Soft Tissue Engineering

**DOI:** 10.3390/ma18071663

**Published:** 2025-04-04

**Authors:** Dawid Łysik, Joanna Mystkowska

**Affiliations:** Institute of Biomedical Engineering, Bialystok University of Technology, Wiejska 45C, 15-351 Bialystok, Poland; j.mystkowska@pb.edu.pl

**Keywords:** compression stiffening, storage modulus, mechanobiology

## Abstract

This study explores the rheological properties of polydimethylsiloxane (PDMS) composites with glass beads (GBs) to replicate the compression-stiffening behavior of biological tissues. The mechanical properties of soft tissues arise from interactions between the extracellular matrix (ECM) and embedded cells. To mimic this, PDMS was used as a polymeric matrix, while rigid GBs acted as non-deformable inclusions facilitating stress redistribution. PDMS composites with 10%, 20%, and 30% GB concentrations were fabricated. Rheological analysis revealed that GBs significantly enhanced the storage modulus (G′), with stiffness increasing linearly under compression. The stiffening rate rose from 300 Pa/% (pure PDMS) to 387 Pa/%, 836 Pa/%, and 2035 Pa/% for 10%, 20%, and 30% GB, respectively, marking a sevenfold increase at the highest concentration. Similarly, the apparent Young’s modulus increased from 150 kPa (pure PDMS) to 200 kPa, 300 kPa, and 380 kPa for composites with 10%, 20%, and 30% GB, respectively. PDMS-GB composites successfully reproduce the compression-stiffening effect observed in biological tissues, which may aid research in mechanobiology and tissue engineering.

## 1. Introduction

Materials that simulate the mechanical behavior of soft tissues are increasingly being utilized in biomedical engineering, spanning from macroscopic tribological studies, which replicate physiological conditions of the stomatognathic system [1], to nanoscale investigations on how environmental stiffness influences cell migration, mechanotransduction pathways, and cancer progression [2].

Polydimethylsiloxane (PDMS) is widely used in biomedical applications due to its biocompatibility, optical clarity, and gas permeability [3]. Its surface exhibits wettability similar to that of soft tissues, while its high elasticity allows it to mimic the mechanics of biological materials. This is particularly relevant considering the wide range of stiffness exhibited by biological tissues and extracellular matrix (ECM) components. For example, collagen fibers range from 100 to 360 MPa; softer ECM proteins such as fibronectin, fibrin, and elastin exhibit much lower stiffness, with Young’s moduli of ≤3.5 MPa, 1–28 MPa, and 1.1–1.2 MPa, respectively [4]. This mechanical diversity reflects the complex nature of biological tissues and the importance of selecting materials for biomimetic applications. A key advantage of PDMS is its tunable mechanical properties, which can be adjusted by modifying crosslinking density and composition; by precisely controlling these parameters, as demonstrated by Heinrichs et al. [5], PDMS elastomers can achieve Young’s moduli as low as 1 kPa, making them suitable for in vitro studies requiring well-defined mechanical environments. This tunability is achieved through various strategies, including adjusting the stoichiometric ratio of crosslinking agents, varying catalyst concentration, incorporating inert polymeric fillers to swell the network, and introducing monofunctional inhibitors to create dangling polymer ends that soften the material. These modifications influence the polymer network structure, reducing the density of elastically active crosslinks and enabling precise mechanical tuning. As a result, PDMS is frequently used in applications ranging from soft tissue modeling to the development of biomaterials that support cell growth and mechanobiological investigations.

A key characteristic of biological tissues is their ability to undergo compression stiffening, where stiffness, defined by the shear storage modulus, increases under compressive loads [6,7,8,9]. In contrast, the extracellular polymeric matrix alone typically softens under compression [10,11]. The compression-stiffening effect in tissues arises from rigid inclusions, such as cells, which redistribute stress and mechanically reinforce the structure. Additionally, poroelasticity effects play a role by regulating fluid movement within the matrix [12,13]. The dominant mechanism behind this effect is the induction of network stresses as cells deform, displace, and rotate under compression. At high cell volume fractions, direct cell–cell contact further amplifies resistance, leading to an additional increase in tissue stiffness [10,12].

Studies on the compression-stiffening effect in tissues have focused on replicating tissue mechanics using simplified systems with a polymer matrix and cells or cell-mimicking elements [14,15]. Hydrogels made from semiflexible biopolymers, such as fibrin or collagen, have been extensively studied and shown to exhibit compression-stiffening effects, whereas synthetic hydrogels like polyacrylamide display weaker responses. Unlike biological tissues, PDMS alone does not undergo compression stiffening, as it lacks the rigid inclusions necessary for stress redistribution under compression. While biopolymer-based hydrogels can partially mimic this phenomenon, PDMS-based elastomers remain largely unexplored in this context.

Rheological studies not only help explain the mechanics of biological tissues but also aim to identify materials suitable for implantology, tissue engineering, and drug testing. Stiffness—particularly the viscoelastic and nonlinear mechanical properties of tissues—plays a key role in regulating essential cellular processes such as adhesion, proliferation, differentiation, and migration [16,17,18]. Cells sense and respond to stiffness variations in their extracellular matrix (ECM) through mechanosensitive pathways, which influence gene expression, cytoskeletal organization, and cell fate [19,20,21]. However, replicating the complex, nonlinear mechanical behavior of biological tissues remains a challenge [22]. Compression stiffening is crucial for maintaining tissue integrity, preventing excessive deformation, and ensuring structural stability [15]. While hydrogel-based approaches offer partial solutions, they often lack long-term stability and fail to reproduce time-dependent mechanical responses, emphasizing the need for alternative materials with both durability and biomimetic mechanical properties.

This study investigates PDMS composites incorporating glass beads (PDMS-GB) to assess their ability to replicate compression-stiffening effects. By examining their rheological properties, we aim to bridge the gap between synthetic and biological materials, contributing to the development of next-generation biomaterials for tissue engineering and regenerative medicine.

## 2. Materials and Methods

### 2.1. Materials and Sample Preparation

The elastomeric matrix was prepared using Ecoflex™ 00-10 silicone rubber (Smooth-On, Macungie, PA, USA), a platinum-catalyzed, addition-cure silicone. The two components (Part A and Part B) were mixed in a 1:1 ratio by weight, following the manufacturer’s recommendations. To reinforce the material, glass beads (Sigma-Aldrich, Saint Louis, MO, USA) were added in specific volume fractions, with their mass determined assuming a density of 2.5 g/mL to ensure a final composite volume matching the intended specimen dimensions. The mixture was thoroughly stirred for homogeneity and degassed under vacuum to remove entrapped air. The material was then cured in a vacuum oven (Steinberg Systems, Berlin, Germany) at 50 °C for 4 h, allowing for complete crosslinking. After curing, specimens were prepared by cutting 20 mm diameter, 2 mm thick disks using a sharp-edged steel punch, ensuring consistent sample geometry. The pure silicone samples were designated as PDMS, while composites containing glass beads were labeled according to their filler content: PDMS + 10%GB, PDMS + 20%GB, and PDMS + 30%GB, corresponding to increasing glass bead concentrations. These samples were used for microscopic evaluation and rheological testing.

### 2.2. CLSM Observations and Measurments

Surface characterization of the samples was conducted using a confocal laser scanning microscope (CLSM) LEXT OLS4000 (Olympus, Japan, Tokyo), employing 10× and 20× objectives to capture high-resolution surface morphology. The specimens were prepared as described earlier and gently cleaned with ethanol before imaging to remove potential contaminants. The laser scanning process was performed using Z-axis scans at 0.1 µm intervals, allowing for precise three-dimensional reconstruction of the surface. To ensure accurate measurements, only minimal surface corrections were applied, including planarity correction to compensate for sample tilt and a pre-measurement filter to reduce noise artifacts while preserving surface details.

For quantitative surface roughness analysis, 10 line profiles were extracted from the most representative surface image, following ISO 21920-2:2021 [23]. Two roughness parameters were measured: arithmetical mean roughness (Ra) and maximum height roughness (Rz). The Ra parameter represents the mean absolute deviation of the surface profile from the central mean line across the evaluation length, effectively quantifying the average texture variation. The Rz parameter is defined as the sum of the absolute height of the five highest peaks and the absolute depth of the five deepest valleys within a given evaluation length, providing insight into extreme surface irregularities.

### 2.3. Rheological Characterization

The rheological properties of the samples were characterized using a Rheostress 6000 rheometer (Thermo Fisher Scientific, Waltham, MA, USA) in a plate–plate configuration. The upper measurement geometry was a serrated 20 mm diameter plate, while the lower plate was also serrated to prevent slippage during testing. Given that the thickness of the prepared specimens varied within ±0.1 mm around 2 mm, the gap was adjusted to ensure a constant normal force of 1 N as the initial contact force before measurements. The temperature was maintained at 37 °C, controlled via a Peltier system to ensure physiological relevance.

Oscillatory tests were performed under two conditions: First, a strain sweep was conducted to measure the storage modulus (G′) and loss modulus (G″) as a function of strain amplitude within the range 0.01–1 at a fixed frequency of 1 Hz. Second, a frequency sweep was performed over a range of 0.1 Hz to 10 Hz at a constant strain amplitude of 0.01. To evaluate compression-stiffening behavior, the compressive strain was varied by adjusting the plate gap in the range of 0–25% with 5% increments, while simultaneously monitoring the normal force to remain within the 50 N force sensor limit. At each compression step, oscillatory measurements were performed for 60 s at a strain amplitude of 0.01 and a frequency of 1 Hz.

### 2.4. Surface Wettability

The surface wettability of the samples was assessed using a Ossila Contact Angle goniometer (Ossila Ltd., Sheffield, UK). The static water contact angle was measured to evaluate the hydrophobic or hydrophilic nature of the PDMS-GB composites. Measurements were conducted three times per sample using precisely dispensed 10 µL droplets of deionized water (Milli-Q quality). The contact angle was determined by analyzing the droplet profile at the solid–liquid-air interface, providing insight into the influence of glass bead inclusions on surface energy and wettability.

## 3. Results

The primary objective of this study was to determine the effect of incorporating non-deformable, rigid structures in the form of glass beads on the properties of PDMS, a polymer widely used for soft tissue simulations. To achieve this, we first characterized the surface morphology of the fabricated composites to confirm the presence and distribution of glass beads. Microscopic analysis revealed that the beads were well-dispersed throughout the PDMS matrix, with their presence becoming more pronounced at higher volume fractions.

**Figure 1:**
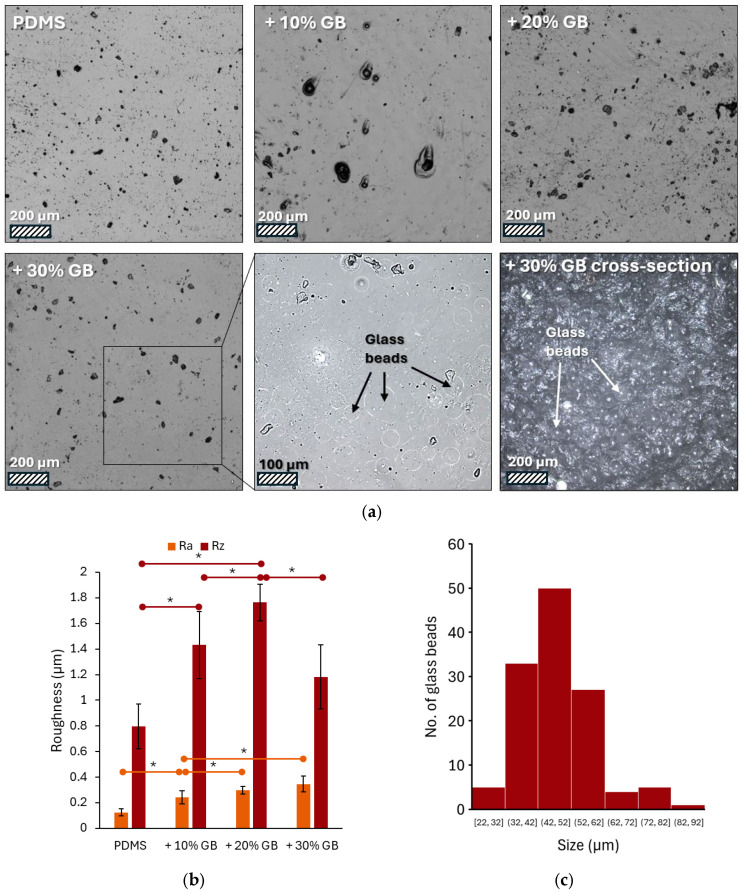
(**a**) Representative laser confocal microscopy images of PDMS surfaces modified with varying volume fractions of glass beads (GBs): pure PDMS, PDMS + 10% GBs, PDMS + 20% GBs, and PDMS + 30% GBs. The presence of glass beads becomes more pronounced with increasing concentration, altering the surface topography. The inset highlights individual glass beads embedded in the matrix. Scale bars: 200 µm (main images), 100 µm (inset). (**b**) Surface roughness analysis (Ra and Rz parameters) showing an increasing trend in roughness with higher glass bead concentrations, demonstrating the impact of rigid inclusions on the PDMS matrix. (**c**) Histogram of the glass beads’ size (diameter) distribution. Asterisks (*) denote statistically significant differences between groups (post hoc Tukey, *p* < 0.05).

Figure 1a presents a series of images captured using laser confocal microscopy, showcasing the surface of PDMS modified with varying volume fractions of glass beads. The pure PDMS surface appears smooth with only minor imperfections, potentially air bubbles. As the concentration of glass beads increases, the surface topography changes, revealing dark spots that are likely protrusions of the embedded beads. These are most prominent at the 30% volume fraction. Due to the partial transparency of PDMS, the microscope focus was set a few micrometers below the surface. This, combined with contrast adjustment, allowed for visualization of the glass bead contours.

The surface roughness of these materials varies, as shown in Figure 1b, which presents the measured Ra and Rz parameters. Analysis of the microscopic images (Figure 1a) revealed an increase in the number and density of glass beads on the surface with increasing concentration in the material. Both Ra (arithmetic average of profile deviations) and Rz (average height of the five highest peaks and five lowest valleys) exhibited a clear upward trend. The addition of 10% glass beads resulted in a 96% increase in Ra and an 80% increase in Rz compared to pure PDMS. At a 20% concentration, these values reached 137% and 121% of the initial values, respectively. The highest concentration of glass beads (30%) led to a 176% increase in Ra. However, the Rz value was 1.1819, representing a 48% increase compared to pure PDMS but a decrease compared to the sample with 20% glass beads. Image analysis showed that the bead diameters ranged from 22 to 92 μm, with an average value of 47 ± 11 μm (Figure 1c).

To further assess the structure–property relationship, we conducted rheological measurements to evaluate the mechanical response of the composites under compression. The introduction of glass beads was expected to influence the viscoelastic behavior of PDMS, particularly in terms of its stiffness response under increasing strain. The findings provide insight into how rigid inclusions impact the compression-stiffening effect, which is crucial in replicating the mechanical properties of soft tissues.

To assess the impact of glass bead (GB) inclusions on the mechanical properties of PDMS, dynamic rheological measurements were conducted under varying strain amplitudes and frequencies. The storage modulus (G′) and loss modulus (G″) were evaluated to understand the viscoelastic behavior of the composites. The obtained results confirm that the presence of rigid inclusions significantly alters the mechanical response of PDMS, leading to increased stiffness and enhanced energy dissipation.

**Figure 2:**
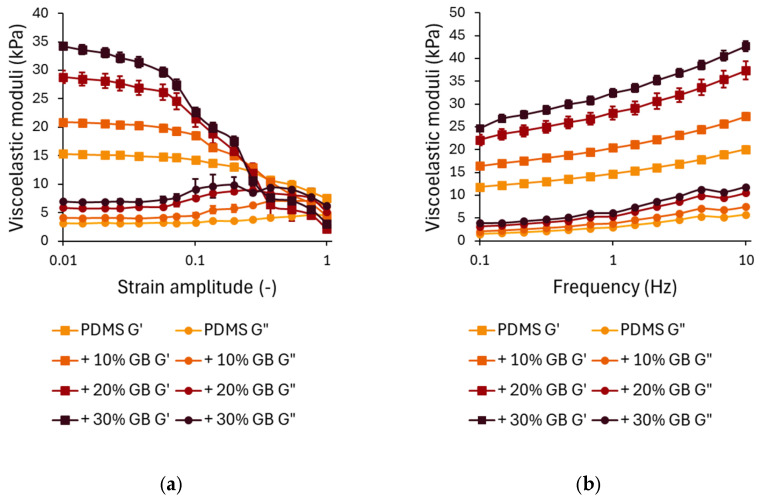
(**a**) Strain-dependent rheological behavior of PDMS composites with varying glass bead (GB) concentrations. Storage modulus (G′) and loss modulus (G″) as functions of strain amplitude, showing a decrease in modulus at higher strains, characteristic of strain softening. The presence of glass beads enhances the initial stiffness. (**b**) Frequency-dependent rheological response of PDMS-GB composites. Storage modulus (G′) and loss modulus (G″) as functions of oscillation frequency, demonstrating increased stiffness and energy dissipation with increasing GB concentration. Error bars represent standard deviations.

The strain-dependent rheological behavior is presented in Figure 2a. In the linear viscoelastic region (LVR) at small strains (<0.1), G′ remains relatively stable, with a clear trend of increasing stiffness as the glass bead concentration increases. Compared to pure PDMS, the storage modulus for 10% GB increased by approximately 40%, for 20% GB by 85%, and for 30% GB by 125%. This stiffening effect demonstrates the ability of rigid inclusions to enhance the network integrity of the composite.

At higher strain amplitudes (>0.1), all samples exhibit strain softening, characterized by a decline in G′. While composites with higher GB content initially show greater stiffness at low strains, they experience a more pronounced decrease in G′ at large strains, with their storage modulus dropping below that of pure PDMS. This suggests that while rigid inclusions reinforce the material at small deformations, they may also contribute to a faster structural relaxation at larger strains, potentially due to interfacial effects or disruptions in the polymer network.

The loss modulus G″ (Figure 2a) follows a similar trend. At small strains, G″ increases with increasing GB concentration, indicating enhanced energy dissipation due to interfacial interactions between the polymer matrix and the inclusions. The peak in G″ at intermediate strains (~0.1) is more pronounced in samples with 20% and 30% GB, suggesting increased internal friction as the polymer chains reorient around the rigid inclusions.

The frequency-dependent mechanical response of PDMS GB composites is shown in Figure 2b. Across the tested frequency range (0.1–10 Hz), both G′ and G″ exhibit a logarithmic increase, characteristic of viscoelastic materials. The frequency-dependent analysis revealed that incorporating GBs significantly enhances G′, particularly at higher loading frequencies. At 0.1 Hz, the 30% GB composite showed a 110% higher stiffness than pure PDMS, emphasizing its increased resistance to deformation. This stiffening effect persisted at 10 Hz, where the 30% GB sample exhibited an additional 112% increase, suggesting that rigid inclusions reinforce the polymer matrix under dynamic loading conditions. This suggests that the incorporation of glass beads significantly reinforces the elastic component of the material. At higher frequencies (10 Hz), the trend is maintained, with G′ for 30% GB reaching 42,667 Pa, an increase of ~112% compared to pure PDMS. Similar trends are observed for G″, where the 30% GB composite demonstrates a ~90% increase in loss modulus at low frequencies and a ~110% increase at higher frequencies compared to pure PDMS. This suggests enhanced energy dissipation due to stress transfer between the glass beads and the PDMS matrix.

The compression-dependent rheological properties of PDMS GB composites were evaluated through storage modulus (G′) and loss modulus (G”) measurements. Under increasing compression, all composites exhibited an increase in G′, confirming the compression-stiffening effect. The pure PDMS sample showed a relatively low stiffening rate, increasing by approximately 52% at 25% compression. However, the 10%, 20%, and 30% GB composites exhibited significant increases of 72%, 91%, and 125%, respectively, demonstrating that the presence of rigid inclusions enhances the mechanical reinforcement of the matrix.

A distinct compression-stiffening effect was observed, with G′ increasing linearly with compression across all composites. Notably, the stiffening rate escalated with GB concentration, reaching 2035 Pa/% in the 30% GB sample, a nearly sevenfold increase compared to pure PDMS. This finding underscores the crucial role of rigid inclusions in modulating the mechanical response.

**Figure 3:**
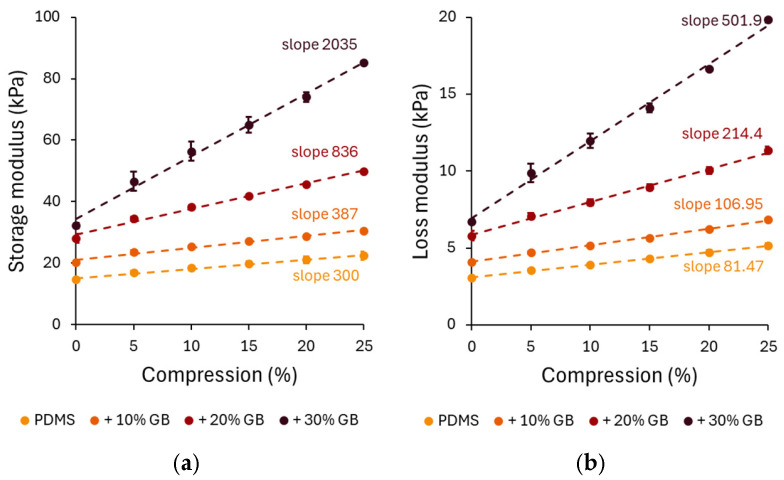

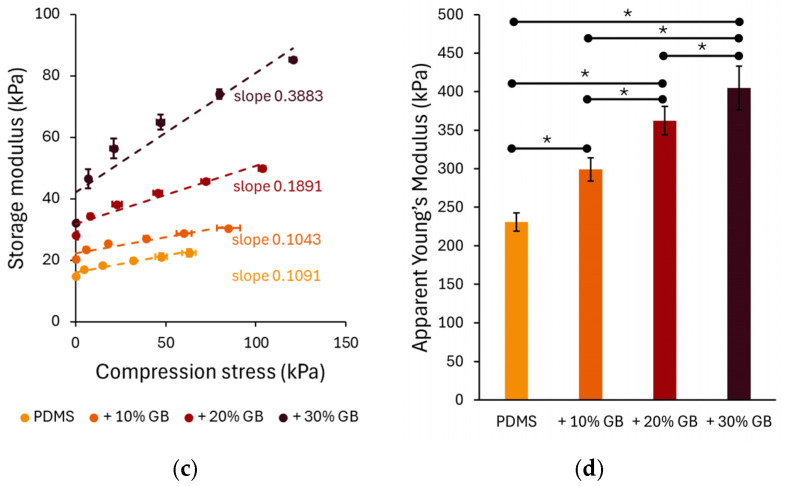
Compression-dependent rheological behavior of PDMS GB composites. (**a**,**b**) Storage modulus (G′) and loss modulus (G″) as functions of applied compression, showing a progressive increase with glass bead content. The slopes indicate the rate of modulus increase, highlighting the compression-stiffening effect. (**c**) Storage modulus as a function of applied compressive stress, revealing a nonlinear stiffening response with increasing stress. (**d**) Apparent Young’s modulus of PDMS composites, demonstrating a significant increase in stiffness with higher glass bead concentrations. Error bars represent standard deviations. Asterisks (*) denote statistically significant differences between groups (post hoc Tukey, *p* < 0.05).

To further analyze the role of stress in compression stiffening, the normal stress (axial stress) was measured during compression and used as the independent variable in Figure 3c. The relationship between normal stress followed a linear behavior, with the slope increasing at higher GB concentrations. The slopes of the linear fits to these data highlight the dependence of the storage modulus on compressive stress, 0.1091 for PDMS, 0.1043 for 10% GB, 0.1891 for 20% GB, and 0.3883 for 30% GB, indicating a stronger reinforcement effect at higher inclusion fractions.

The apparent Young’s modulus (Figure 3d), derived from the slope of normal stress vs. compression strain curves, also showed a strong dependency on GB concentration. The Young’s modulus increased from 230 kPa in pure PDMS to 299 kPa (+10% GB), 362 kPa (+20% GB), and 404 kPa (+30% GB), confirming the progressive reinforcement effect of rigid inclusions on the composite’s elastic properties.

**Figure 4:**
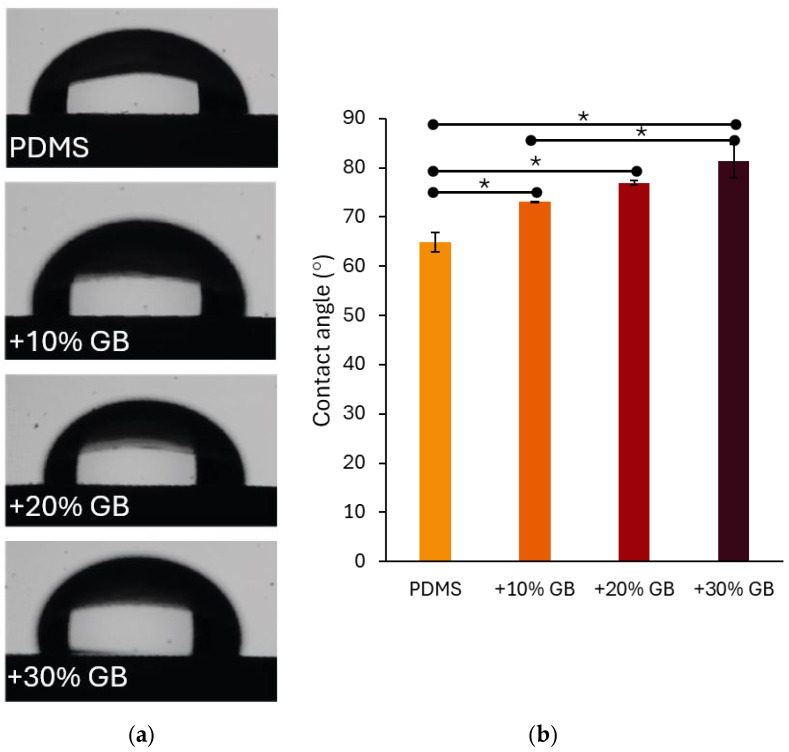
(**a**) Contact angle measurements of water droplets on PDMS surfaces with varying glass bead (GB) concentrations. The presence of glass beads alters the surface wettability, increasing hydrophobicity. (**b**) Quantitative analysis of contact angle values for pure PDMS and composites with 10%, 20%, and 30% GB content. The contact angle increases with higher GB concentrations, indicating a significant shift in surface properties. Asterisks (*) denote statistically significant differences between groups (post hoc Tukey, *p* < 0.05).

In addition to mechanical reinforcement, surface wettability is a crucial characteristic of biomaterials, particularly in applications involving cell cultures. The contact angle measurements presented in Figure 4 indicate that the incorporation of glass beads into PDMS significantly modifies its hydrophobicity. The contact angle increased from 64.87° ± 1.99° for pure PDMS to 73.15° ± 0.06° for 10% GB, 76.98° ± 0.40° for 20% GB, and 81.43° ± 3.43° for 30% GB. The observed increase in contact angle, reaching 81.43° for the 30% GB composite, suggests a transition toward greater hydrophobicity. This shift is particularly relevant for biomedical applications, as hydrophobic surfaces may reduce non-specific protein adsorption while influencing cell adhesion dynamics and bio-interface interactions. The observed trend suggests that the presence of rigid inclusions not only impacts bulk mechanical properties but also alters the physicochemical characteristics of the material surface, potentially affecting interactions with biological environments. This finding reinforces the hypothesis that PDMS GB composites can serve as effective mechanical models for tissue-mimicking applications in biomedical engineering.

## 4. Discussion

This study confirms that incorporating rigid inclusions, in the form of glass beads, induces compression stiffening in PDMS composites, an effect absent in pure PDMS. This behavior closely resembles not only biological tissues containing cellular inclusions [7,8,24,25,26], where stress redistribution enhances mechanical resistance under compression, but also biofilms, which exhibit similar reinforcement mechanisms due to the presence of embedded microorganisms and rigid inclusions in extracellular polymeric substances [27,28]. The stiffening effect scales with glass bead concentration, as reflected in the increasing storage modulus (G′) under both oscillatory shear and compressive loading. Compression-dependent rheological analysis demonstrated a linear increase in G′ with compression, with the 30% GB composite exhibiting the highest slope (2035 Pa/%), consistent with theoretical models describing composites with rigid inclusions in a compliant matrix [10].

While many biopolymeric networks exhibit shear stiffening, biological tissues typically undergo shear softening, where G′ decreases with increasing shear strain [8,24]. The PDMS-GB composites also demonstrate shear softening, distinguishing them from strain-stiffening polymeric gels but aligning them with soft biological tissues such as the liver, the brain, and fat. This response suggests that stress redistribution by the inclusions weakens shear resistance, similar to how cells interact within the extracellular matrix (ECM). In contrast, compression stiffening, which is prominent in fibrous tissues like cartilage and muscle, arises due to cell–matrix interactions that enhance mechanical stability under compressive loads. The behavior of PDMS-GB composites suggests that they replicate both compression stiffening and shear softening, making them a promising biomimetic material.

The compression-stiffening effect can be interpreted within classical elasticity frameworks, particularly the Barron–Klein (BK) and Birch models [6]. The BK model describes anisotropic stress redistribution, predicting that G′ increases linearly with axial stress, with slopes between one-quarter and one-half. Birch’s theory, in contrast, applies to fluid-filled structures, where compression induces hydrostatic stiffening, yielding slopes between 1 and 4.5. The experimental data align more closely with the BK model, with slopes increasing from 0.1091 (pure PDMS) to 0.3883 (30% GB), indicating that stress remains partially anisotropic rather than fully hydrostatic. This suggests that PDMS GB composites mimic fibrous ECM-rich tissues like cartilage rather than fluid-dominated organs such as the brain.

Young’s modulus (E), a measure of material stiffness, was determined from compressive stress–strain relationships, revealing a progressive increase with GB concentration: 230 kPa for pure PDMS, rising to 404 kPa for 30% GB. These values place the composites within the stiffness range of muscle and soft cartilage (10 kPa–1.5 MPa) [29,30,31,32] but far below tendons (0.5–1.2 GPa) or bone (10–20 GPa) [33,34]. Unlike the standard Young’s modulus, the apparent Young’s modulus accounts for heterogeneous stress distribution in composites [35,36], where rigid inclusions act as local reinforcements. This effect mirrors how biological tissues achieve mechanical integrity through cellular and ECM interactions.

The frequency-dependent rheological response further supports the role of rigid inclusions in enhancing dynamic mechanical stability. As frequency increased from 0.1 Hz to 10 Hz, pure PDMS exhibited a 70% rise in G′, while the 30% GB composite showed a 110% increase, indicating improved resistance to cyclic loading. This behavior is significant for biomedical applications, where materials must endure repetitive mechanical stress, such as in soft tissue scaffolds, implants, and load-bearing biomaterials [16]. The concurrent increase in the loss modulus (G″) suggests that GB inclusions also enhance energy dissipation, a critical factor in preventing mechanical fatigue in applications like cartilage replacements and soft-tissue prosthetics.

Beyond mechanical properties, surface wettability is a crucial factor influencing biocompatibility, particularly in protein adsorption and cell adhesion [37]. Contact angle measurements revealed a progressive increase in hydrophobicity with GB concentration, from 64.87° (pure PDMS) to 81.43° (30% GB). This shift suggests that rigid inclusions alter the polymer’s surface energy, potentially affecting cell–material interactions. Moderate hydrophobicity can reduce non-specific protein adsorption while still supporting cell attachment, making PDMS GB composites tunable for tissue engineering scaffolds, biosensors, or drug delivery platforms.

In summary, PDMS GB composites exhibit compression stiffening, shear softening, and tunable mechanical properties, closely replicating soft biological tissues. Unlike hydrogels, which degrade over time, PDMS-based materials offer long-term durability with customizable stiffness, making them promising candidates for biomedical scaffolds, implant coatings, and biomechanical modeling. Future research should explore viscoelastic modifications and anisotropic reinforcement strategies to further refine their biomimetic potential.

## 5. Conclusions

Our study demonstrates that compression-stiffening effects can be engineered in PDMS-based elastomers through the addition of rigid inclusions, offering a promising approach for developing tunable biomaterials applicable in tissue engineering, soft robotics, and mechanobiology. The results indicate that increasing the glass bead (GB) concentration enhances the material’s mechanical properties, particularly in terms of stiffness and viscoelastic response.

However, the presented study has certain limitations. The initial spatial distribution of glass beads within the polymer matrix has not been thoroughly analyzed, nor has their potential rearrangement under shear and compressive loading. Understanding how these inclusions migrate or rotate during mechanical testing could provide further insights into the observed compression-stiffening effect. Additionally, in the current model, the glass beads are virtually undeformable and significantly stiffer than living cells, which deform in response to applied stresses. This difference may influence the degree to which the presented material mimics the mechanics of biological tissues.

Another limitation is the lack of investigation into the effect of inclusion shape on compression stiffening. While spherical beads were used in this study, biological cells and extracellular matrix components exhibit more complex morphologies that may impact their mechanical interactions with the surrounding matrix. Moreover, living cells possess the ability to chemically or physicochemically interact with their extracellular environment, a factor that is not accounted for in the PDMS-GB system. These aspects should be explored in future research to enhance the biomimetic potential of the material.

Future studies should focus on optimizing the composition of PDMS composites to achieve even more biologically relevant mechanical properties. Advanced imaging techniques, such as micro-computed tomography (µCT) or confocal microscopy, could be employed to characterize the three-dimensional distribution of inclusions and their behavior under mechanical loads. Additionally, further investigations should explore alternative fillers with tunable stiffness and deformability, enabling better replication of cell–matrix interactions. Lastly, biological validation through cell culture experiments would be a crucial step in assessing the material’s suitability for biomedical applications.

In summary, PDMS GB composites exhibit promising properties that make them relevant for soft tissue modeling and biomaterial development. Addressing the identified limitations and extending the scope of research will further enhance their applicability in mechanobiology and regenerative medicine.

## Data Availability

The data presented in this study are available on request from the corresponding author.
